# Dr. Elliotson on Hydrocyanic Acid, in Affections of the Stomach, Lungs, &c. &c.

**Published:** 1821-03-01

**Authors:** 


*Cases illustrative of the Efficacy of the Hydrocyanic or Prussic Acid in Affections of the Stomach, with a Report upon its Powers in Pectoral and other Diseases in which it has been already recommended, and some Facts respecting the Necessity of varying the Doses of Medicines according to Circumstances, and the Use of Opium in Diabetes.* By JOHN ELLIOTSON, M.D. &c. &c. One vol. 8vo.—1820.

THE adaptation of the various parts of the material world to our use is an admirable illustration of that benevolence which the Supreme Being exercises towards his sensitive and rational creatures. This truth, at all times conspicuous, becomes eminently so in an æra, when the penetrating search of the philosopher is continually developing a salutary tendency in those powers, which are wont to be regarded as the very bane of our nature. A variety of disorders, which seemed almost beyond the reach of medicine, have, within these few years, yielded to the skilful management of the most virulent poisons. And heartily do we congratulate every practitioner of the healing art, that to this class of remedies another is now added, which, we confidently believe, will be found inferior to none that have preceded it.

With the *prussic*, or (as it is now called) the *hydrocyanic** *acid*, every chemist must have been long familiar. Its pernicious effects on the animal body were first pointed out in a series of experiments performed by Dr. Madden, about the year 1730.† Soon afterwards, Dr. Browne Langrish instituted a number of trials, with the view of determining the medicinal qualities of this deleterious fluid. Such attempts however proving nugatory, the prussic acid underwent little farther scrutiny, until Doctors Majendie and Granville, about three years ago, again subjected it to the test of medical investigation. Their reports of its use in pectoral complaints were so favourable, as to induce the author of the little volume before us, to have recourse to it in his own practice; and, fortunately for mankind, here it was that the remedy happened, by mistake, to be administered to a person labouring under severe dyspepsia. The rapid amendment consequent upon its exhibition, excited a strong suspicion in the mind of Dr. Elliotson, that so striking a change had resulted from the employment of the acid. He resolved, therefore, on ascertaining the truth of this conjecture, by trying it on an extensive scale. An accumulation of *very valuable facts*, decisive of the accuracy of such an opinion, was the consequence. These form the bases of Dr. E's essay. They are divided into sets. The first set, comprising seventeen cases, is intended to illustrate the power of the acid over that affection of the stomach, which is indicated by *pain and tenderness in*

---

* The disposition which there exists to obscure the science of medicine by the invention of high-sounding names, cannot be too severely reprehended. Even the chemists, whose predilection for hard words is notorious, saw enough of technicality in the title "prussic," to be satisfied. Why, then, should the sons of Æsculapius feel so desirous of a change? Is it (which is their only excuse) that they fancied they had found another term more descriptive of the thing signified? If so—grievously we apprehend have they erred in judgment. For, granting that every one understood the language from which the word is derived, and that no inconvenience would arise from its extraordinary length, (both of which suppositions are inadmissible) even then we deny that the adjective κυανεος, serves so well to denote the exact species of blue resulting from the trifling combination of acid, alkali, and iron, as that epithet, which, by a reference to the very substance itself, must immediately recal the precise idea of colour, if originally excited in the mind.

† These experiments were indeed performed with *laurel water*; but, as no doubt is now entertained of the identity of its active ingredient with Prussic acid, they have been adverted to as fair illustrations of the virtues of the latter fluid.

*the epigastrium simply*. Ten of the persons thus afflicted were females. Generally, they had attained to the middle, or rather the advanced periods of life; and the duration of their complaint varied from a few weeks or months, to as many years. But, notwithstanding its almost inveteracy, it gave way to the use of the acid. In the majority of instances, indeed, the cure was effected in a few days or weeks; in none was it protracted so long as two months. We pass on then to the second set, in which we are presented with ten cases of pain and tenderness, accompanied by the usual symptoms of *dyspepsia, as flatulence, vertigo, head-ache, loss of appetite, nausea, vomiting, debility, nervousness, cough, and dyspnœa*. Here, also, the acid proved equally successful, although the complaint had existed months, and, in some instances, even years. The control, in short, it manifested over these multifarious symptoms, would alone afford a sufficient demonstration of its extraordinary influence in stomach derangements. But, satisfactory as must be this conclusion to every unprejudiced mind, it will receive yet further confirmation from the five cases included in Dr. E's third division. These were examples, differing in no respect from those just mentioned, except in their greater degree of severity, and in being marked by an additional symptom, (viz.) *the pyrosis or watery secretion of the stomach*. No one can be ignorant how apt is this obstinate species of indigestion to baffle the endeavours of the most skilful. But, even here, our remedy did not fail: for in three of the five cases specified, it soon effected a perfect cure; and, so remarkable was the improvement in the remaining two, that, had the medicine been duly persisted in, complete success would, doubtless, have followed.

Instances of dyspepsia, characterized by the frequent *recurrence of vomiting*, are by no means uncommon. To elucidate the efficacy of the acid over this modification of the disorder, eight cases are succinctly given, which, though of considerable standing, were all, in no long time, removed.

In the very brief analysis we here attempt of Dr. E's treatise, it is incumbent on us not to overlook *a circumstance*, which might be considered as affecting the validity of his conclusions. On a few occasions, he prescribed (he informs us) *a gentle laxative* in conjunction with the acid. It is possible, therefore, the good ascribed by him to this latter remedy might, with greater justice, have been attributed to its concomitant. Anticipating however this objection, the Doctor has obviated it by the introduction of five other cases, which constitute his fourth division. In the first of these the patient progressively recovered, his bowels being, all along, in a constipated condition; in the second, scarcely any amendment took place for a considerable time, though they were kept relaxed by appropriate medicines; in the third, fourth, and fifth cases, complete restoration ensued, notwithstanding the most obstinate costiveness. Hence, then, it is obvious, that the particular state of the intestinal canal possesses very little influence, either in promoting or retarding the salutary operation of our remedy on the stomach. At any rate, its power of restoring the healthy functions of this organ, does not appear to be diminished by the *existence of fœcal accumulations*. With what shadow of probability, then, can the sanative process in question be ascribed to the *laxatives?* These reflections bring us to Dr. E's sixth and last division, which, although it include only four cases, is, we apprehend, one of no ordinary importance. It states the efficiency of the acid, in that formidable species of dyspepsy, which often *counterfeits diseases of the heart*. So exactly do the more prominent features of these opposite affections correspond, that we confess it has occurred to us, to mistake the one disease for the other. The vehement and rapid pulsation in many instances of stomach ailments, accompanied, as it often is, by acute pains darting from the sternum and left breast, down the spine and left arm, together with the benumbed sensation of this member, and the hurry and dejection expressed in the countenance, are calculated to mislead even the most wary. But, in the prussic acid, we have an excellent method of discrimination: for, should they appear altogether beyond its control, they may fairly be presumed to be connected with some structural derangement of the circulating organ.

Such, then, is Dr. E's experience with regard to the virtues of this remedy in *functional disorders of the stomach*. Highly extolled as it has been in a variety of pectoral complaints, it did not, in the hands of our author, appear to deserve the encomiums it has received. He found it, indeed, serviceable in simple dry cough, and the spasmodic asthma; but in pneumonia and hooping cough, it seemed altogether useless. In phthisis it produced a slightly soothing effect, and that only occasionally. In hæmorrhages, in palpitations of the heart from organic derangements, and in all other disorders where it is desirable to diminish the force of the blood's motion, our author apprehends the prussic acid will prove of little avail. Several hysteric, epileptic, and maniacal patients, took it without the smallest benefit; but it presently wrought a cure in the only case of chorea, to which it was applied. In rheumatism it proved totally inert, but there is some ground for supposing it possessed of anthelmintic qualities. In the proportion of one or two drachms to a pint of water, it has appeared to allay the irritation of the prurigo pudendi and some other cutaneous affections.

From this description of its virtues, we proceed, briefly, to notice our author's *method of exhibiting* so potent a remedy. With persons of adult age, he generally began its administration in doses of a single minim, thrice repeated in the course of twenty-four hours; and, provided neither nausea nor giddiness (which are its first sensible effects) nor other inconvenience ensued, he gradually augmented the quantity to two, three, and even to six minims. "Almost any adult (observes Dr. E ) will bear one or two minims, few more than five; three are generally borne and required, and very frequently four. One woman took xvii minims three times a day without inconvenience or benefit: xviii brought on vomiting and giddiness." To the youngest infant may be given a quarter of a minim, (i. e.) half a drop, one minim being nearly two drops. An over-dose will produce vomiting, pain and tightness at the stomach, with fainting; and if the quantity be immoderately large, convulsions and death. The only instance of its fatal effects on the human subject, with which we are acquainted, is recorded in the very interesting trial of Captain Donellan, for the murder of Sir Theodosius Boughton. The body of this unfortunate gentleman had passed too far into a state of putrefaction, to admit of any accurate anatomical deductions. But the effects of laurel water on the inferior orders of animals, as detailed by several physicians produced at that trial, are worthy of an attentive perusal. In some of their experiments, death instanly took place; and, in all, so speedily, as to render the supposition of its being the consequence of previous inflammation in the stomach wholly inadmissible: whilst, on the other hand, the frightful convulsions which this poison, in adequate doses, never failed to excite, clearly prove that its operation is, primarily and principally, on the nervous system. This supposition will go a good way towards explaining the modus operandi of the prussic acid, in severe dyspeptic cases. No sooner is it swallowed, than the pain accompanying such states often ceases. From this moment the vitiated secretions shew evident signs of amendment, and go on progressively improving, until they are, at length, subdued. Now, as the formation of healthy gastric juices evidently depends on *a regular transmission of nervous influence into the secerning organ*, it may fairly be presumed, that any *deviation* from such *supply* would cause this depravation. If, then, our remedy be supposed to possess the power of correcting all irregularities in the distribution of this subtle fluid upon the stomachic nerves; or, in other words, of restoring the balance of excitability at this part, we might conclude, a priori, that its operation would be speedy, and its effects just what observation teaches us. Quitting however speculation, we are anxious to fix the minds of our readers upon considerations of much greater importance—actual facts.

Unlike many other powerful agents in medicine, the prussic acid may be taken for an indefinite period, without incurring the hazard of an accumulated operation. But, it must be at the same time admitted, that there are constitutions with which it does not seem to agree. The inconvenience, however, in such cases, is only temporary, and by no means of a serious nature. Upon the bowels, it has a slightly constipating effect, and, occasionally, it augments the discharge of urine. It will be borne in the middle and after parts of the the day better than at first rising; and, should any feeling of coldness exist in the stomach, it may be advantageously combined with aromatic and bitter infusions.

As the process usually adopted in the preparation of our remedy involves much chemical skill, it will sometimes fall to the lot of practitioners to use a spurious and ineffectual sort. This precaution is the more necessary, as such accident must materially tend to the discredit of a medicine, which, when genuine, will be found to be of great value. We deem it, therefore, incumbent on us, to point out to our readers those indications which, in our opinion, are decisive of its purity.* The fluid should be colourless and perfectly transparent, powerfully emitting the odour, and leaving on the tongue the flavour of bitter almonds. Having been at considerable pains to determine its specific gravity, we can safely assert that at the temperature of 60°, it is to that of distilled water at the same temperature, as .9931 is to 1. Should the liquor appear turbid or deposit any sediment, its genuineness may be suspected. This change diminishes its activity, and is, in all probability, produced by the absorption of oxygen, which converts it from a ternary compound (consisting of hydrogen, carbon, and azote) to a quaternary, which is composed of these elements with the oxygen superadded. We have only further to remark on this topic, that, as the active principle of which we here speak is a gazeous vapour, held in solution by water, easily decomposed by light and driven off by heat, it must be kept in a dark and cool situ-

* At the recommendation of Dr. E. we obtained the medicine at Mr. Garden's the Operative Chemist, 372, Oxford Street.

ation. The method best adapted for general purposes is an earthen-ware vessel, filled with water, and covered over.

Thus much with regard to the prussic acid. We now proceed to notice the *two remaining sections* of Dr. E's volume. The former of these consists of a few cases illustrative of that power, with which the body, in certain morbid conditions, *resists the ordinary influence of remedies.* And, although we are fully sensible of the value of such instances in a pathological point of view, yet we cannot but regard the extreme minuteness with which they are detailed, as quite unnecessary for all practical uses.

The first was a case of spasmodic dyspnœa, in which the paroxysms, though recurring almost daily, were regularly subdued, and at length permanently removed, by prodigious doses of opium. Only sixty drops of the tincture were at first administered, but this proportion, in four days, was increased to ʒvi, and in eight, to ℥j, with no other effect than that of giving the patient some comfortable sleep.

The second case, was an instance of *insanity*, accompanied by great heat and throbbing of the head, and obvious disorder of the chylopoietic viscera. It was treated with *calomel*, at first, *in eight grain doses*, but afterwards *in doses of ʒiss.* In six months *nine ounces* of this potent mineral were actually administered, and that too without originating any other inconvenience, than ordinarily ensues from the regular and moderate use of it; and, although it did not ultimately eradicate the disorder, the proofs of its controlling power were always manifest.

Dr. E. next presents us with five instances of the admissibility of enormous doses of pulvis antimonialis in hemiplegic, convulsive, and other nervous disorders. By some of these individuals, *sixty*, *seventy*, *and even eighty grains*, were taken thrice in the course of twenty-four hours, for several successive days, *unattended by any unpleasant symptom*, *save a slight nausea*, *or*, *occasionally*, *a gentle disposition to vomit.* By this extraordinary fact, Dr. E. thinks the long litigated point, as to the identity of the pulv. antimon. with James's powder, might be decided. For, should a similar administration of the latter substance act with violence in similar cases, this circumstance, he apprehends, would at once prove the diversity of their nature.*

* We are probably much in the dark yet respecting the *extent* to which medicine may be carried with perfect safety. In Italy, where pathology and therapeutics are now cultivated with unequalled zeal, the sulphas ferri is given, with great success, in dropsical affections con-

The only remaining section of Dr. E's work relates to a very interesting subject, (viz.) *the efficacy of opium in the diabetes mellitus*. This important fact, though obscurely hinted at by some preceding writers, was first distinctly noticed by that eminent practitioner, Dr. Darwin. To his testimony of the advantage of such a method, are subsequently added those of Drs. Ferriar and Warren, and Mr. Money, a surgeon at Northampton, now of London. But, to our author it is that we are indebted for any thing like a statistical and satisfactory account of its effects in this disorder. From his statements it is manifest, that the free use of opiates is not only capable of *restraining the enormous secretion* of fluid which takes place in diabetes, but, also, of *divesting it of that saccharine principle*, with which it abounds; and of ultimately restoring it to a condition, in every respect, natural. To effect this desirable purpose, however, it was necessary to administer the medicine in prodigious doses. Sometimes ℈i, at others ʒss, and in one of the detailed cases, not less than ℈ii of solid opium were actually given three times a day, for upwards of a fortnight. At the recital of these facts, some of our readers may perchance be a little startled, but to ourselves, who have repeatedly had occasion to witness the extreme apathy of diabetic patients, both mental and bodily, they are not at all surprising.

It is the remark of certain practical writers, that diabetes mellitus is *always* preceded by a state of urine differing from the healthy, only in respect of quantity. It happening to our author, therefore, to meet with such cases of *increased secretion*, he regarded them as the preludes of that disorder; and accordingly treated them with opiate medicines, to which they readily yielded. It appeared also from the analysis of that able chemist, Dr. Prout, that the urine, in these cases, contained by far too great a proportion of urea. Such considerations, corroborated as they are by the near resemblance of this substance to the saccharine principle, led Dr. E. to the supposition, that the sweetness of diabetic urine is simply the effect of a depravation of its chief saline constituent, the urea. The precise nature of the change, it would be difficult perhaps to determine; but, as the addition of azotic gas to

---

nected with, or dependent on, inflammatory affections of the serous membranes, and that in doses which we would scarcely credit. They begin with five grains twice or thrice a day, and increase it to one or two drachms for a dose. Instead of augmenting febrile symptoms, it lowers the pulse, increases the urine, and all the secretions and excretions, thus removing the dropsy, and improving the constitution, at the same time. *Ed.*

the elements of which this singular substance is composed, is known to give sugar, the Doctor throws out this merely as a conjecture, whose reasonableness, indeed, appears to us yet further confirmed by the reflection, that in the process of secretion there is ground to believe, that the circulating mass is resolved, by the various organs of the body, into its elementary parts; and by them again so combined, as to form the different secreted fluids.

Such, then, is the brief outline of Dr. E's publication, which, though ushered into the world in an humble form, and with a diffidence which ever accompanies true merit, will be found to be a valuable addition to our medical literature. Candour, talent for observation, and accuracy of discrimination, are conspicuous in the work throughout; and what he has advanced on the subject of prussic acid, we have ourselves, in the main, *confirmed and verified* by a pretty extensive scale of experiment.* The first proof we had of its efficacy, (which was indeed the first trial we gave it) was in a case of severe dyspepsy, which had for three months resisted the combined influence of alterative and bitter tonics, and was accompanied by a most violent and continual palpitation of the heart. The instantaneous relief it afforded, at once astonished and delighted us. The pain in the epigastrium ceased, and the troublesome symptoms rapidly declined until they had all (including the alarming action of the heart) in the space of a week, entirely disappeared. And, to pass over many other instances equally convincing, we would, for a moment, direct our reader's attention to the case of a poor man, who had for some years past, at distant intervals, been subject to *convulsive attacks*. He had had three fits the day he visited us, and was actually seized again in our presence. It remained upon him about ten minutes, during which time the arms were agitated upwards and downwards incessantly, the head drawn towards one side, and the muscles of the face violently distorted. He informed us, a painful distension of the epigastrium always warned him of their approach; and that he, almost constantly, experienced a degree of soreness and fluttering at that part. Thus directed to the stomach as the "fons et origo" of his affliction, we prescribed for him the acid three times a day in minim doses. Immediately after the first dose (which was administered without delay) the epigastrium was relieved,

* The Editor entrusted the review of this work to a physician in the country, whose public practice at a charitable institution, and private experience qualified him to report practically and usefully on the publication.

and, although a month has now elapsed since we first saw him, the fits have never recurred. For ten days past he has been occupied in his usual employment, and with the exception of a slight uneasiness, once or twice, transiently felt at the stomach, he has remained entirely free from complaint.

These considerations, together with our own experience of its inutility, in inflammatory and structural affections, will not suffer us to withhold from Dr. E. the merit of having ascertained and defined, by a chain of judicious experiments, the more important medicinal qualities of the prussic acid. The forcible manner in which he has demonstrated its operation to be, specifically, on the stomach, has, indeed, suggested to us an application of the remedy, which our author does not seem to have contemplated. We allude to its use in those painful disorders, gout, gravel, and diabetes, which the best modern writers agree in ascribing to derangements in the digestive functions. In a severe calculous case, which recently came under our care, it was given with the decided effect of allaying both vomiting and pain; and we have sanguine hopes its efficacy may also extend to those other "opprobria medicinæ," at which we have just hinted. At any rate, we are resolved on omitting no fair opportunity of determining this point, and we trust our brethren in the profession will deem the subject of sufficient interest to institute similar inquiries. From such an investigation much important knowledge, we are persuaded, would result. Perhaps it would disclose to us new paths of research. But, whilst we augur thus favourably, let it not be imagined that we belong to that class of visionaries who, on the discovery of any valuable medicine, never rest until they have ruined its reputation by proclaiming it a panacea. We are of opinion, that the prussic acid will be found possessed of little efficacy except where the stomach is concerned. Many instances of dyspepsia, which spring from depraved hepatic or other chylopoietic secretions, we are aware, will never yield to it. But, whenever the disorder consists in, originates from, or is connected with, derangements in the *gastric functions*, (excepting however those cases in which the structure also of the organ has become morbid) then will the remedy evince its amazing powers. How ample, therefore, the sphere of its operation!

